# Are chemical compounds in medical mushrooms potent against colorectal cancer carcinogenesis and antimicrobial growth?

**DOI:** 10.1186/s12935-022-02798-2

**Published:** 2022-12-01

**Authors:** John M. Macharia, Lu Zhang, Ruth W. Mwangi, Nora Rozmann, Zsolt Kaposztas, Tímea Varjas, Miklós Sugár, Huda Alfatafta, Márton Pintér, Raposa L. Bence

**Affiliations:** 1 Doctoral School of Health Sciences, Faculty of Health Science, University of Pẻcs, City of Pẻcs, Hungary; 2 Medical School, Department of Public Health Medicine, University of Pẻcs, City of Pẻcs, Hungary; 3 Doctoral School of Horticultural Sciences, Institute of Vegetables and Mushroom Growing, Hungarian University of Agriculture and Life Sciences, Budapest City, Hungary; 4 Faculty of Science, Department of Biological Sciences, Egerton University, Nakuru City, Kenya; 5 Faculty of Health Sciences, University of Pécs, City of Pécs, Hungary

**Keywords:** Colorectal cancer, Bioactivity, Medicinal mushrooms, Antimicrobial properties, Antibacterial, Antifungal

## Abstract

After cardiovascular diseases, cancer is the second main cause of death globally. Mushrooms have been demonstrated to contain amalgamation with properties capable of inhibiting carcinogenesis and microbial growth, principally secondary metabolites such as quinolones, steroids, terpenes, anthraquinones, and benzoic acid derivatives among others. This study aimed to substantiate their potency concerning colon cancer carcinogenesis and antimicrobial growth. A systematic search of important literature was performed considering all the articles published until April 2022. Screening was performed by searching the BMC Springer, Elsevier, Embase, Web of Science, Ovid, and MEDLINE databases. In addition, Google Scholar was used to supplement information. Titles and abstracts that matched the established criteria were selected for full-text article scrutiny and subsequently used in the updated present review. Bioactive compounds present in medicinal mushrooms such as ascorbic acid, organic acids, flavonoids, polysaccharides, glycosides, phenols, linoleic acid, grifolin, and tocopherols among other compounds play a key role in suppressing the proliferation of cancerous cells and selectively act as antibacterial and antifungal agents. These metabolites actively scavenge oxygen free radicals, hydroxyl radicals, and nitrite radicals that would otherwise increase the risks of the growth and development of cancerous cells. Mushrooms' bioactive compounds and metabolites actively inhibit nuclear factor-kappa activation, protein kinase B processes, and ultimately the expression of Cyclooxygenases 2 in cancerous cells. Medicinal mushrooms should be considered as alternative natural chemo-preventive agents in the global fight against colon cancer and the evolution of drug-resistant pathogenic microorganisms, as they exhibit robust potency. They have not been reported to exhibit adverse harmful effects compared to synthetic chemotherapies, yet they have been reported to demonstrate significant beneficial effects.

## Introduction

### Mushrooms characteristics and colon cancer intervention

Mushrooms have been demonstrated to contain amalgamation with properties capable of inhibiting microbial growth, principally secondary metabolites such as quinolones, steroids, terpenes, anthraquinones, and benzoic acid derivatives [1]. They also constitute substantial primary metabolites such as proteins, peptides, and oxalic acid [1]. The pharmacological activities of these mushrooms are associated with their polysaccharide constituents displaying antitumor, antibacterial, and anticancer potency [2]. Research linked to transcriptomics studies and genomics of mushrooms of medical importance like *Lignosus rhinocerotis* has contributed to a better understanding of their molecular biology and expanded avenues for more investigative studies extending to the genome level [3]. Genome expressions in medicinal mushrooms portray substantial pharmacological ability significant to human health [3].

After cardiovascular diseases, cancer is the second main cause of death [4]. Localized tumors with limited growth are medically described to be of a benign characteristic while those that spread to other parts of the body and are aggressive on healthy tissues are said to be metastasized and of a malignant characteristic [4, 5]. Colorectal cancer is largely associated with cancer-related deaths among western cultures and is currently ranked 3rd [6]. Chemotherapeutic interventions and surgery are the commonly employed forms of intervention for colorectal tumors for lack of alternative intervention strategies. The exploration, development, and identification of effective bioactive molecules capable of eliminating cancerous cells without killing normal cells or being hazardous to normal cells is of significant medical impact [7]. For this reason, management using dietary supplements derived from plants is starting to gain the attention as the most effective method of reducing the burden of colon cancer-associated mortality [8]. Essentially, the strategy used to prevent colon tumors is dependent on approaches towards diagnosing adenomatous polyps that are precursors for colon cancer [9]. Edible mushrooms have important biomolecules that are essential benefits for growth and development in virtually all living organisms. They are largely consumed as a food source and for their medicinal values [10]. However, their pharmacological properties and efficacy are poorly understood. Most phytochemicals with determined bioactive potential have been associated with plants [10].

Mushrooms are taxonomically classified as Basidiomycetes and some as Ascomycetes. They provide nutrients including easily digested proteins, fibre, minerals, carbohydrates, vitamins, and antioxidants. They are therefore largely consumed because of their nutritional benefits as cited by [1]. Further, the authors observed that it was not easy to distinguish between the edible mushrooms from medicinal mushrooms because most of the commonly available edible ones have medicinal properties while other identified species with medical properties are equally edible [1].

There still exists a dire need to develop new, affordable, and effective anticancer drugs [4]. More than 35,000 plant species have been evaluated for their medical significance and this has resulted in the discovery and development of anticancer medicines such as Indicine–N-oxide, Vinblastine, Taxol, Etoposide, Vincristine, Camptothecin, analogues, and other numerous drugs [4, 5].

### Influence of diet and lifestyle on tumor biology

Although certain cancers have a genetic predisposition, the major external causes of this disease have been linked to lifestyle choices, exposure to pollutants in the environment, and dietary [11]. Due to exposure to new environmental influences, adaptation to different lifestyles, and diets, this is one of the plausible explanations why country variations in the frequency of some types of cancer vanish in respective immigrant communities [11, 12]. According to the National Academy of Sciences in the USA, dietary variables are thought to be responsible for 40% of all male cancers and 60% of female cancers [13]. Chemical tumorigenesis is a multiphase process that takes place over a considerable amount of time. A myriad of interactions between genomes, the environment, and cellular metabolism are involved during this incredibly complex process [14]. Numerous nutrients can be combined in a beneficial way to prevent tumor growth, limit cancer cell invasion, and reduce angiogenesis and metastasis [15]. Additionally, nutritional synergy has been shown to be successful in inducing the apoptotic process in a variety of cancer cell types [15, 16]. Behavioural (lifestyle) factors like lack of physical exercise, smoking, and excessive alcohol consumption, all contribute to high incidence of malignant tumours [17]. CRC risk is reported to increase with cigarette smoking history [18]. Whereas smoking, which may account for 20% to 30% of all incident cancers, is unquestionably the most significant lifestyle-related risk factor overall, it is closely followed by consumption of alcohol and obesity. Studies have demonstrated that tobacco smoke carcinogens can cause base substitutions that are linked to cancer, such as G: C to A: T transitions in *RAS* oncogenes [18, 19]. However, a number of sizable investigations have shown that smoking was more closely linked to incident CRCs that were *KRAS* mutation-negative than *KRAS* mutation-positive [18, 19]. However, the significance of particular risk factors for various cancer types and subtypes varies considerably [20]. In Western nations, clinical outcomes suggest that the major environmental and lifestyle factors tend to be responsible for 40%-60% of cancer cases, which strengthens the potential of primary prevention [21].

### The significance of diet and lifestyle on human gut microbiome

The modulation of the composition and metabolic activity of the human G.I tract microbiota, which has an influence on health, is becoming widely understood to be a function of dietary (specifically macronutrients) and other environmental factors [22]. In comparison to the number of somatic cells in the body, there are roughly 100 trillion more bacteria in the human gastrointestinal (GI) tract. The gut can also contain yeasts, single-cell eukaryotes, viruses, and tiny parasitic worms in addition to the majority of the microorganisms, which are bacteria [23]. The greatest strategy to maintain a healthy gut microbiota population may be through dietary measures, notably the usage of a variety of fibre. Approaches like consuming probiotics and prebiotics can help to maintain microbial balance and subsequently improve human health [22]. In spite of the fact that many dietary polyphenols may have biological effects via anti-oxidant or anti-inflammatory pathways, polyphenols that infiltrate the colon can be degraded by the intestinal bacteria and generate bioactive products [24, 25]. The colonic microbiota's fermentation of fibre and the metabolites that are created as a result are responsible for many of the health benefits associated with it. Organic acids produced during the fermentation of carbohydrates give other bacteria, the gut epithelium, and auxiliary tissues energy [22, 26]. The primary by-products of carbohydrate fermentation are short chain fatty acids (SCFA). These weak acids (pKa 4.8) help decrease the pH of the colon, which prevents growth of pathogenic bacteria [26].

It is with increased dependence on synthetic colon cancer treatment options that this paper undertook to review mushrooms of medical importance containing natural bioactive agents with potency against colon cancer growth and proliferation. Plants' natural constituents may provide a new source of anticancer and antimicrobial treatment with a sufficient novel mode of action. Compared to synthetic agents, phytoconstituents of plant origin are rarely seen to correlate with numerous side effects and have been demonstrated to present overwhelming therapeutic activities to heal numerous infectious diseases [5]. Medicinal mushrooms will play a critical role in colon cancer prevention and as a potential antimicrobial agent.

## Methods

### Electronic databases and search output

Screening for important literature was duly performed by searching the BMC Springer, Elsevier, Embase, Web of Science, Ovid, and MEDLINE databases. In addition, Google Scholar was used in supplementing information. Articles in google scholar were first screened for authenticity by interlinking the various articles identified with their corresponding existing publisher. The search yielded a total of 3805 papers that were published between the years 2000 and April 2022. As a result of not meeting the inclusion criteria, a total of 3685 articles were excluded from the study. A total of 120 articles met the inclusion criteria and have adequately been discussed in the present review.

### The screening criterion and search strategy

For the study, the authors first independently screened only English-language publication titles and abstracts from primary studies, considering all the articles published until April 2022 [27, 28]. Research on malignancies other than CRC and studies involving plants that were highly poisonous to cells and had adverse effects were technically excluded from the study [8]. After being considered appropriate by 3 independent authors, titles and abstracts that met the set criteria were recruited for full-text article scrutiny and subsequently used to provide the necessary analytical data for the present review. Titles and abstracts that matched the established criteria were selected for full-text article scrutiny and subsequently used to supply the necessary analytical data for the present review after being deemed appropriate by three independent writers. Authors' independence was essential when determining whether or not to use the enrolled articles in order to eliminate potential bias risks [8, 28].

“Colorectal cancer”, “adenomatous polyps”, “colon cancer”, “colon tumor”, “colorectal tumor”, terms were then combined with either of the following MeSH terms: “Bioactivity”, “biological activity”, “anti-cancer”, “phytochemicals”, “pharmacological activities”, “anti-tumour”. These terms were further combined with either of the following terms “mushroom”, “medicinal mushroom”, “bacteria”, “anti-bacterial”, “antifungal, antiviral”, or “anti-microbial”.

## Anti-colorectal cancer properties

The burden of colon tumor pathogenesis is quite multiplex and not well understood. However, its initiation process has been associated with the interactive effects of peril factors like lifestyle, heredity, and environmental factors [29, 30]. Empirical treatment and management of colon carcinoma include the use of immunotherapy, chemotherapy, radiotherapy, cytotoxic drugs, surgical and resection procedures, and targeted therapy [31–33]. Oxygen-free radicals, hydroxyl (OH) radicals, lipid peroxidation, and nitrate radicals (nitrosamines) increase the risks of growth and development of cancerous cells [34]. Yang et al. [35] cited that OH radicals could inflict severe harm to close cellular cells within the body leading to apoptosis and consequently the development of cancerous cells.

Bioactive compounds present in medicinal mushrooms such as ascorbic acid, organic acids, flavonoids, polysaccharides, glycosides, phenols, tocopherols among other compounds [36, 37] play a key role in suppressing the proliferation of cancerous cells by scavenging on these free radicals. Medicinal mushrooms have also been identified to constitute substantial amounts of ergocalciferol (vitamin D2). Hasnat et al. [38] observed in their experimental study that the extracts of *Russula virescens* exhibited substantive scavenging effects on OH radicals in a dose-dependent mechanism. Derived extracts of *Hohenbuehelia serotina, Dictyophora indusiata*, and *Hypsizygus marmoreus* have also been established to demonstrate very significant scavenging potential towards OH radicals, with potency levels reaching 100% [39]. Phytoconstituents are important molecular compounds common in many herbal plants as well as in medicinal mushrooms and are critical in promoting good health. They largely influence advancements in medical science, and research in particular since they have important beneficial properties [1]. Grifolin is a natural bioactive constituent present in *Albatrellus confluence* and has been cited to be an important bioactive anticancer agent. It has been demonstrated to inhibit the growth and aggression of cancerous cells by suppression of the ERK1/2 pathway and by induction of apoptosis [40]. Bioactive compounds present in medicinal mushrooms could prevent colon carcinogenesis by modulating biochemical activities in the gastrointestinal gut (GIT) [41].

Na et al. [42] examined the in vitro and in vivo anticancer effects and probable mechanisms of sporoderm-broken spores of *Ganoderma lucidum* (*G. lucidum*) water extract (BSGLWE) on colorectal cancer. According to their findings, BSGLWE considerably reduced the viability of colorectal cancer HCT116 cells in a dose- and time-dependent fashion. According to flow cytometry research, BSGLWE interfered with the advancement of the cell cycle at the G2/M phase by downregulating cyclin A2 and cyclin B1 while as well it upregulated the P21 at the level of mRNA. Additionally, BSGLWE caused apoptosis by lowering the protein levels of PARP, Bcl-2, pro-caspase-3, and pro-caspase-9 as well as the mRNA levels of survivin and Bcl-2 [42].

Additionally, through an in vivo experiment, BSGLWE inhibited tumor growth by controlling the expression of genes and proteins linked to cell cycle and death. This effect was further supported by a decrease in Bcl-2, PCNA, and Ki67expression as shown by immunohistochemical labelling [43]. The pro-apoptotic gene NSAID activated gene-1 (NAG-1) was markedly increased by BSGLWE therapy at both the mRNA and protein levels, both in vitro and in vivo. Additionally, BSGLWE caused an increase in the relative concentrations of NAG-1 secreted in both mouse serum and cell culture medium therapies, indicating a part for NAG-1 in the anticolorectal cancer action brought on by BSGLWE [43].

Similarly, Li et al. [44] investigated triterpenoids obtained from ethanol extracts of sporoderm-broken spores of *G. lucidum*. Overall, they reported that BSGLEE efficiently prevents colorectal cancer carcinogenesis by promoting cell cycle arrest, inducing apoptosis, and inhibiting migration. Based on the suppression of apoptosis by reversing microtubule polymerization, Li et al. [45] hypothesized that *G. lucidum* polysaccharide could mitigate intestinal barrier impairment caused by paclitaxel. *G. lucidum* polysaccharide, one of the most studied and representative polysaccharides, is regarded as a prebiotic candidate due to its anti-tumor action [46]. The benefits of *G. lucidum* polysaccharide for host cancer prevention, particularly CRC, have been shown in numerous trials.

Additionally, a thorough understanding of how the gut microbiota and *G. lucidum* polysaccharide interact has been developed. Using CRC mice as a model, Luo et al. [47] discovered that consumption of *G. lucidum* polysaccharides (GLPs) could significantly alter CRC symptoms by promoting the relative abundances of Enterobacteriaceae, Bacteroides, and Peptostreptococcaceae and lowering those of Desulfovibrionaceae, Oscillospira, Clostridiales and Ruminococcus [47]. Guar gum, which was discovered to enhance the presence of Akkermansia with *G. lucidum* polysaccharide intake, was also found to be less helpful at alleviating CRC symptoms [45].

Another study established that the combination of jiaogulan saponins and *G. lucidum* polysaccharide could significantly reduce CRC-associated symptoms, including the tumor cell proliferation, inflamed gut barrier, and production of oncogenic signalling molecules, which are connected to the rise in relative abundances of Bacteroidetes and other SCFAs-producing bacteria [28].

In another different study, Dan et al. [48] described the isolation of a protein from *G. lucidum* that demonstrated anticolorectal cancer properties and purified the ribonuclease protein, (17.4-kDa) using liquid chromatography techniques. They reported that the ribonuclease protein could arrest the cell cycle at G1 phase by controlling the expression of cyclin D1 and P53 in HCT116 and HT29 colorectal cancer cell lines. The *G. lucidum* ribonuclease protein demonstrated strong anti-proliferative and anti-colony formation actions. The ribonuclease was shown to activate pathways controlled by caspase9 and the unfolded protein response to cause cell death in HCT116 cells. Additionally, the ribonuclease treatment drastically reduced the capacity of HCT116 cells to engage in autophagy, a stress adaptation mechanism to deal with metabolic crises [48].

Jeff et al. [49] reported the anti-colorectal cancer benefits of polysaccharides obtained from mushrooms. They investigated the polysaccharide fractions from *Lentinus edodes* (WPLE-N-1, WPLE-N-2, and WPLE-N-3) in vitro and their results showed to have anti-proliferative activities against HT-29 and HCT-116 cells. They further reported that the in vitro proliferation assays for adherent HT-29 and HCT-116 carcinoma cells and suspended S-180 sarcoma cells indicated that the three water-soluble polysaccharides had a higher antitumor activity against suspended cells than adherent cells [49].

Polysaccharides from certain Termitomyces mushrooms have anticancer effects. Both water-soluble and insoluble β-glucan obtained from hot water extracts of *T. robustus* showed immunomodulatory qualities by significantly stimulating thymocytes, splenocytes and macrophages.

## Cyclooxygenase-2 inhibitory potency

Cyclooxygenases are proteinous and are correlated with the production of lipid prostaglandins. They are significant for biochemical processes in the body [41]. Cyclooxygenases 1 (COX-1) facilitates the process of homeostasis by modulation while Cyclooxygenases 2 (COX-2) are of importance in inflammatory processes as an immunoregulatory response [50]. In small amounts, COX-2 is expressed in the large intestine, but it can also be regulated by cytokines, growth factors, tumor necrosis factors, and lipopolysaccharides under duress. High amounts of COX-2 are associated with the growth and proliferation of colorectal tumors [41, 50].

Crude ethanolic extracts (50 μg/mL) of *Elaphomyces granulates* inhibited the COX-2 mechanism of action by about 68%, while the present bioactive compounds: syringic acid and syringaldehyde inhibited the COX-2 mechanism in a dose-dependent manner, with an IC_50_ of 0.4 μg/mL and 3.5 μg/mL, respectively [51]. Terpenoids obtained from *C. hookeri* and *Inonotus obliquus* caused downregulation of iNOS and COX-2, and inhibition of mRNA expression of iNOS and COX-2 respectively also. In addition, Agaricoglycerides derived from *Grifola frondose* induced upregulation of NF-κB and the production of COX-2, iNOS, TNF-α, ICAM-1 and IL-1β [51]. Numerous phytochemicals have been established from the fruiting bodies of *Grifola frondosa*. The most significant among these are polysaccharide fractions which stimulate the immune-competent cells and enhance antitumor activities [52].

Preventive synthetic COX-2 has been cited to have harmful side effects prompting the exploration of natural inhibitors as alternative forms of chemoprevention interventional therapies in colorectal cancer management. Natural antioxidants abundant in mushrooms such as polyphenols and carotenoids (Table 1), suppressively prevent the oxidation of lipids, proteins, and nucleic acids and consequently influence the initiation of oxidizing chain reactions [53]. Of great medical concern, is that synthetic antioxidants such as butylatedhydroxyanisole and butylatedhydroxytoluene have recently been demonstrated to be carcinogenic and cause adverse side effects [53].

**Table 1:** Bioactive ingredients/compounds in medicinal mushrooms active against colorectal carcinogenesis

Mushroom Species	Bioactive ingredients/Compounds present	Type of activity	References
*Agaricus bisporus*	*p*- polysaccharides, hydrobenzoic acid derivatives, Gallic acid, phenols, Pyrogallol, Flavonoids, Tocopherols,	Reducing power, scavenging of superoxide radicals, lipid peroxidation inhibition	[1, 56]
*Agaricus comtulus*	Phenols	Bleaching inhibition of β-carotene	[57]
*Agaricus campestris*	Phenols	Reducing potential	[1, 58]
*Agaricus lutosus*	Phenols	β-carotene bleaching inhibition	[57]
*Agaricus romagnesii*	Phenols	Reducing potential, inhibits lipid peroxidation	[59]
*Antrodia camphorata*	γ-tocopherol, diterpenes, polysaccharides, ascorbic acid, phenols	Inhibits lipid peroxidation	[60]
*Boletus edulis*	Phenols e.g., flavonoids, organic acids, polysaccharides	Reducing potential, lipid peroxidation inhibition	[1, 61]
*Cantharellus cibarius*	Polysaccharides, flavonoids, pyrogallol	Scavenging for potential	[62]
*Cordyceps Sinensis*	Exopolysaccharides, polysaccharides, protein complexes	Reducing potential	[63]
*Calvatia gigantean*	Polyunsaturated fatty acids, calvacin, phenolic compounds	Antioxidative potential	[64]
*Flammulina velutipes*	Polysaccharides, Phenols, Flammulin	Lipid peroxidation inhibition, scavenging OH radicals, β-carotene bleaching inhibition	[65]
*Hericium erinaceous*	Steroids, phenols, erinacines, mono-terpenes, diterpenes	Lipid peroxidation inhibition, scavenging for free radical	[64]
*Lentinus edodes*	Polysaccharides, phenols chitosan, *p*-hydroxybenzoic acid, lentinan, glucan, mannoglucan	Scavenging for OH radicals, prevents lipid peroxidation	[66, 67]
*Lignosus rhinoceros*	Polysaccharide-protein phenolics,	Activity against superoxide anion radical	[68]
*Morchella esculenta*	Phenols, polysaccharide, β-1,3-D-glucan, galactomannan, heteroglycan	Scavenging for OH radicals, inhibit lipid peroxidation	[67, 69]
*Pleurotus eryngii*	Polysaccharide-protein complex, laccase, heteropolysaccharide acid	Scavenging against hydroxyl radicals	[70]
*Pleurotus ostreatus*	β-carotene, cinnamic acid, α-tocopherol, flavonoids, phenols	Scavenging potential against superoxide, OH radicals and prevents lipid peroxidation	[71]
*Pleurotus sajor-caju*	Phenols	Scavenging for free radical and lipid peroxidation prevention	[72]
*Polyporus squamosus*	Tocopherols, phenols	Scavenging potential for radicals	[73]
*Polyporus tenuiculus*	Phenols	Scavenging for DPPH radical, Chelating ability for Ferrous ion	[74]
*Russula delica*	Catechin, phenols, tocopherols	Chelating for Ferrous ion, inhibition for β-carotene bleaching, scavenging for OH & superoxide radicals	[75]
*Verpa conica*	Phenols, tocopherols,	Scavenging for superoxide radical	[76]
*Volvariella volvacea*	Phenolic compounds	Scavenging for OH radicals, inhibit lipid peroxidation	[77]

Signalling pathways like mitogen-activated protein kinase (MAPK), phosphatidylinositol 3-kinase (PI3K), nuclear factor-kappa B (NFκB, and protein kinase B (AKT) modulates the expression of COX-2. The influence of the NFκB process is mediated by P13K via AKT [54]. Mushrooms' bioactive compounds and metabolites (Table 1), actively inhibit NFκB activation, AKT processes, and ultimately the expression of COX-2 in cancerous cells [54, 55].

## Production of polysaccharides by different mushrooms

### Lentinan polysaccharide

**Fig. 1:**
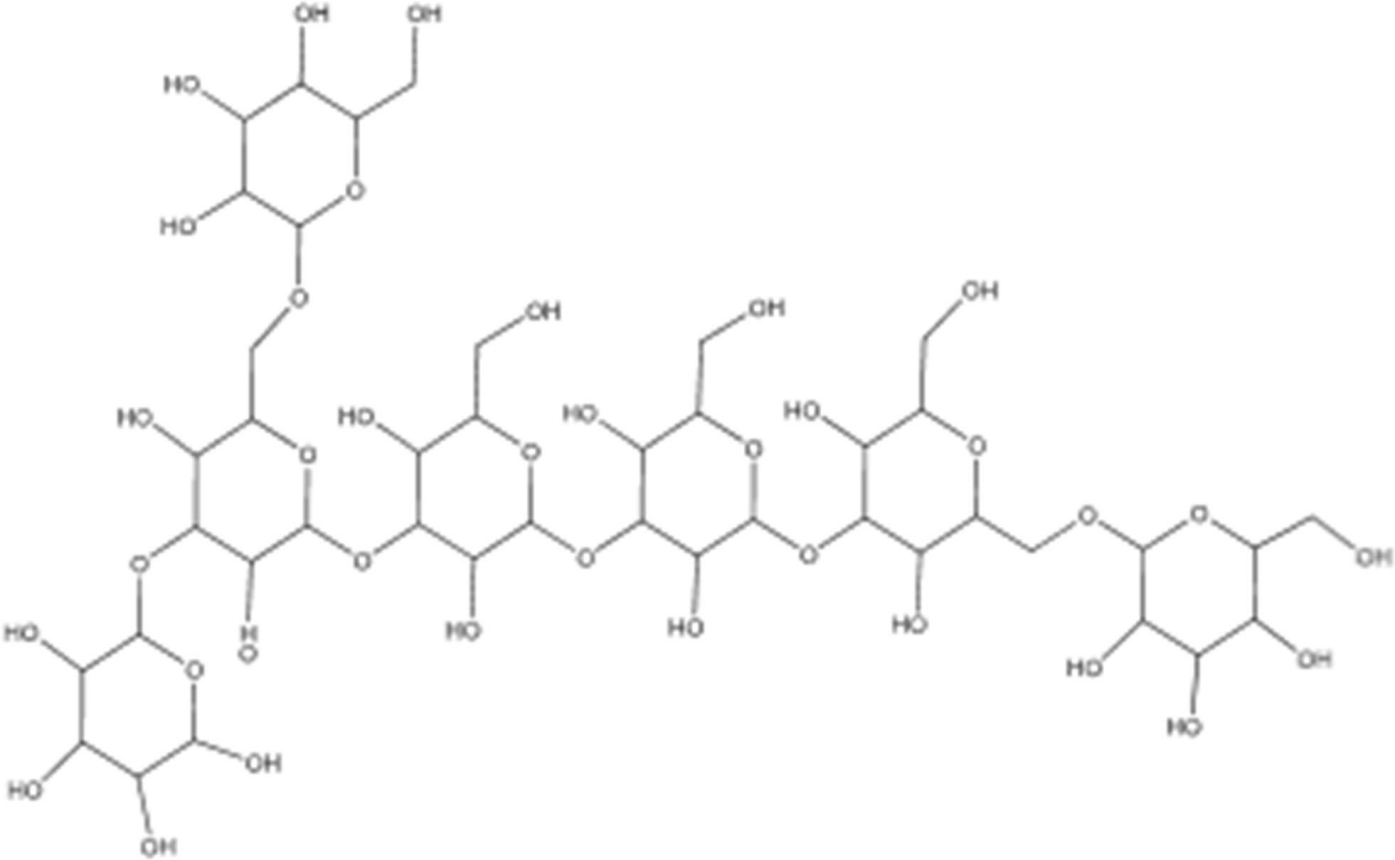
Lentinan

Lentinan (Fig. 1) is a polysaccharide with beta-glucans and displays effective immune potentiating and antitumor activity. It is produced by *Lentinus edodes* and additionally demonstrates immunomodulating properties through the release of cytokines from immunocytes, and therefore are suitably utilized in prevention of different cancers. Lentinan acts as an intravenous anti-tumor polysaccharide [78].

By acting directly on macrophages or indirectly through lentinan-stimulated T cells, lentinan causes an increase in the production of a number of bioactive serum factors linked to immunity and inflammation. These include IL-1, IL-3, CSF, vascular dilation inducer, and acute-phase protein induction, which results in the induction of numerous immunobiological changes in the host [78].

### Polysaccharide-K, (PSK/Krestin)

**Fig. 2:**
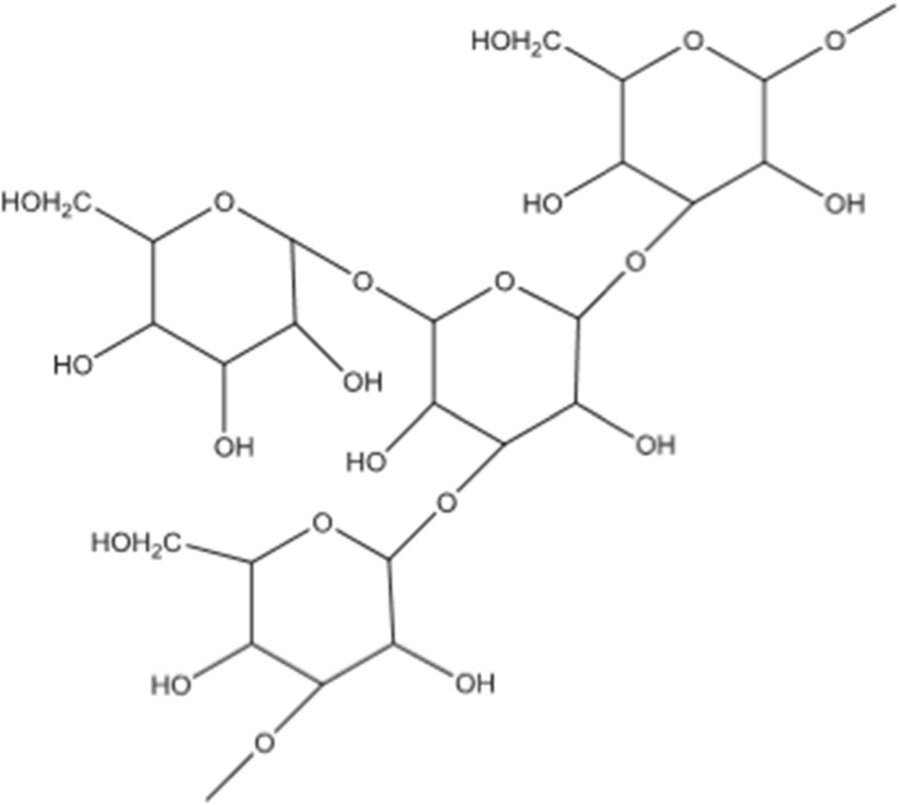
PSK/ Krestin

This is a proteoglycan found in the polypore fungus *Trametes versicolor* and is made up of proteins and β-glucans with 25–38% protein residues. Along with neutral amino acids like leucine and valine and trace levels of basic amino acids like arginine and lysine, it primarily comprises acidic amino acids like glutamic and aspartic acids. Krestin (Fig. 2) is a distinct protein-bound polysaccharide that has been employed in the treatment of various cancers (pancreatic, lung) as a chemoimmunotherapy agent [79, 80].

### Maitake D-fraction polysaccharide

**Fig. 3:**
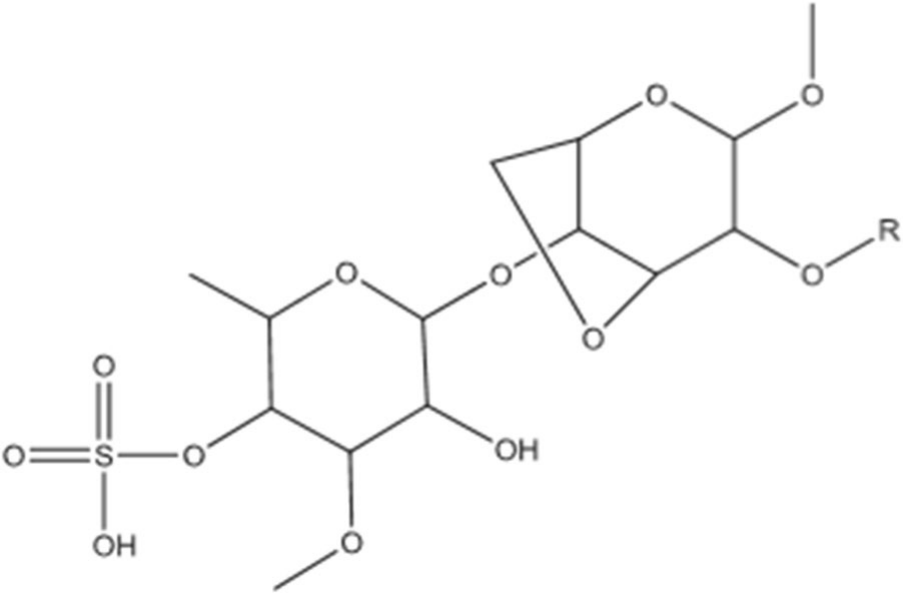
Maitake D-fraction Polysaccharide

The protein-bound polysaccharide (proteoglucan), composed of β -glucan, or maitake D-fraction (Figure 3), is a bioactive extract of the maitake mushroom (*Grifola frondosa*) [81]. In patients receiving immunotherapy and chemotherapy concurrently, maitake D-fraction has been shown to diminish the growth of hepatic, lung, and breast malignancies [81, 82]. The fraction alone reduced expression of tumor markers, prevented metastasis, and increased natural killer (NK) cell activity [71, 81, 82].

### Schizophyllan polysaccharide

**Fig. 4:**
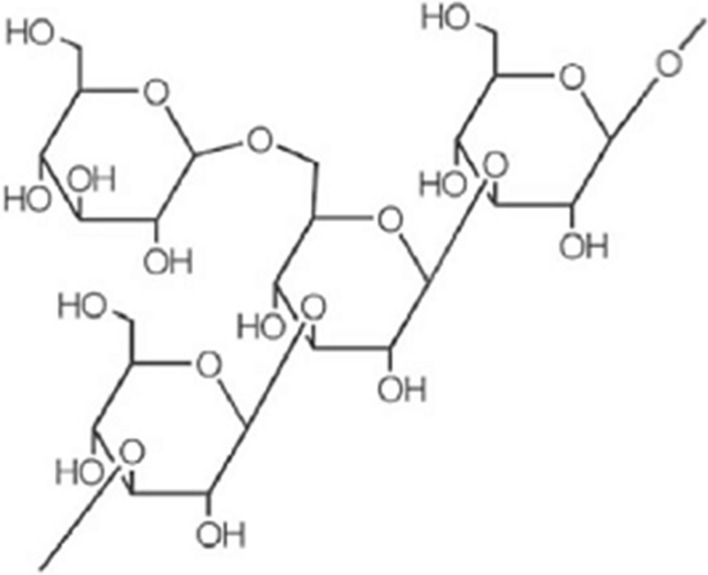
Schizophyllan polysaccharide

*Schizophyllum commune* produces schizophyllan polysaccharide (Figure 4). It is comparable to lentinan in terms of chemical structure, which refers to the makeup of sugars and how they are linked. Cancers of the stomach neck and the throat are treated with this medication [83]. Due of its radioprotective qualities, it is also given during radiation. Schizophyllan revives bone marrow cells' previously reduced ability to undergo mitosis [84].

## Antibacterial properties of mushrooms

Endophytic microorganisms that inhabit the tissues of plants are rarely explored through research although they are significant potent sources of novel naturally occurring products for exploitation in medical research, agricultural intervention, and in processing industries [85, 86]. Fungi are largely the most significant isolated endophytic microorganism with established secondary metabolites with identified antibacterial and antifungal activity [85]. Focused research and the development of effective antibiotics are still very significant due to the continuous occurrence of multi-drug-resistant bacterial pathogens [86].

Urgency is needed to curtail and control increasing antimicrobial drug resistance by use of improved antibiotic therapy and minimize hospital cross-infection [86]. In this regard, various mushrooms have been identified to have antibacterial effects. For example, *Flammulina velutipes, Polyporus squarnosus, Hericium erinaceus, Psathyrella sp, Tricholoma sp, Lentinus edodes, Trametes sp,* and *Agaricus bisporus* [1] have all been evaluated and given satisfactory inhibition against bacterial growth. Research studies have shown that fermented mushroom mycelia constituting black rice bran or supplemented with turmeric actively inhibited the growth of *Samonella typhimurium* (*S. typhi*) [87]. The mechanism of antibacterial activity demonstrates that phagocytosis against *S. typhi* in the macrophage cells and antibacterial activity in albino mice is correlated with increased autophagic-macrophage activity, causing macrophage-mediated antibacterial mechanism and a systemic antibacterial activity via type 1 interferon [87].

The antibacterial characteristics of extract recovered from the fruiting body of *Coriolus versicolor* (*C. versicolor*) were observed in a different study against *Staphylococcus aureus* (*S. aureus*) and *Salmonella enterica*. The Minimum Inhibitory Concentration (MIC) levels of the tested bacteria ranged from 0.625 to 20 mg mL^-1^. *C. versicolor* demonstrated noteworthy activity against both Gram-negative and Gram-positive bacteria. The growth curves of *Salmonella enterica and Staphylococcus aureus* measured at 630 nm, and precisely confirmed with a macrodilution method demonstrated that the recovered *C. versicolor* methanolic extract actively inhibited the growth of the two bacterial strains tested in the study [88]. Other extensive studies done have also demonstrated that *Bacillus subtilis, S. aureus,* and *Bacillus cereus* are largely inhibited by methanolic extracts from *Boletusedulis*, *Agaricus bisporus* and *Cantharelluscibarius* mushrooms [89]*.* Further research has similarly established that the antibacterial activity of extracts obtained from *Agaricus blazei* mushroom exhibited significant inhibition for most Gram-positive bacteria compared to the Gram-negative bacteria tested. The extracts inhibited bacterial strains of *Streptococcus pneumoniae, Salmonella typhi, Streptococcus mutans, P. aeruginosa, Streptococcus sobrinus, S. aureus, Listeria monocytogenes, B. cereus,* and coliforms [90]. The mechanism of inhibition is associated with structural differences common in Gram-negative bacteria such as a thin peptidoglycan layer and numerous flow pumps [90]. Linoleic acid present in these medicinal mushrooms has been identified to be an important bioactive molecular compound that facilitates the antibacterial mechanism and activity of these mushrooms [91]. A study conducted in Kenya further points to the effectiveness of mushrooms collected in the wild against bacterial growth. The investigated mushrooms in the study included *Trametes* spp. and *Microporus* spp. and demonstrated activity against *Escherichia coli, Klebsiella pneumoniae, Pseudomonas aeruginosa, Pseudomonas aeruginosa, and S. aureus* [92].

[93] reported that Bacillus cereus, Yersinia enterocolitica, Vibrio parahaemolyticus, Listeria monocytogenes, Clostridium perfringens and Staphylococcus aureus, were all shown to be inhibited by an aqueous extract of Lentinus edodes mushroom. In contrast, it had no effect on E. coli, which is consistent with the results obtained by. This mushroom was reported to exhibit greater antimicrobial potential against gram positive bacterial as contrasted to gram negative bacterial [94].

*Ganoderma pfeifferi* and *G. australe* extracts have been demonstrated to have potent antibiotic activity against *E. coli* [95, 96]. Methanolic extract of *G. lucidum* demonstrated exceptional antibacterial activity against *B. subtilis, E. coli,* and *Salmonella* species. Disk diffusion studies have demonstrated that the chloroform extract of *G. lucidum* have growth-inhibiting effects on *B. subtilis* and *S. aureus* [97].

The MIC value for *G. australate* extract against *P. aeruginosa* and *E. coli* was 2 mg/ml, whereas it was 0.25 mg/ml for *Bacillus* species and 1 mg/ml for *S. aureus* [96]. A high MIC of an aqueous *Ganoderma* extract against *B. subtilis* (3.5mg/ml) and *Bacillus* species (3.5mg/ml) was also reported. *Staphylococcus, Streptococcus, Escherichia,* and *Vibrio* species are susceptible to Polyperin, which was identified from *Polystichus sanguineus* [93]. Numerous gram-positive and gram-negative bacteria can be attacked by steroid extracts from *Ganoderma applanatum* [98]. The antibacterial action of *Lentinula edodes* against *S. aureus* and other bacteria has been attributed to the presence of oxalic acid [99].

## Antifungal properties

To exist and survive in their natural ecosystems, mushrooms must generate antifungal and antibacterial biomolecules [100]. In this regard, fungicidal metabolites with active potency have been isolated from medicinal mushrooms and could be of significant beneficial effects to man [101]. Fungal bioactive compounds can be acquired from many sources including cultivated fruiting bodies, wild, supernatant of submerged cultured by the use of bioreactors, or from mycelial biomass [102].

Unlike ethanolic and chloroform extracts, hot water extracts have exhibited the most powerful antifungal activity. The hot water extraction method yields numerous antimicrobial compounds including terpenoids, flavonoids, and tannins [103]. This activity is correlated with the fact that most antimicrobial active biomolecules such as terpenoids and flavonoids are seemingly polar and cannot be sufficiently extracted using a less polar solvent such as chloroform. Besides, hot water efficiently penetrates inside the intracellular matrix of the cell walls [92, 103]. *Trametes* spp. and *Microporus* spp. extracts have been reported to actively inhibit *Candida albicans, C. tropicalis,* and *C. parapsilosis* fungi of medical importance [92]. It has further been demonstrated that *A. bisporus, A. bitorquis, A. essettei,* and *Oudemansiella canarii* methanolic extracts exhibited substantial activity against *Candida* spp. Grifolin, isolated from *Albatrellus dispansus* mushroom appeared to be the most significant bioactive molecular compound against the tested phytopathogenic fungi [100]. *Ganoderma lucidum (G. lucidum)* methanolic extracts showed an activity MIC=0.005mg/mL against *Trichoderma viride* [100]*.* Other authors have further demonstrated that water extracts and ethyl acetate with 5% DMSO of *Climadocon pulcherrimus, Agrocybe perfecta*, *Pycnoporus sanguineus,* and *Oudemansiella canarii* exhibited substantial antifungal potency against *Candida krusei* [104].

The low molecular weight terpene compound grifolin exhibits overwhelming antifungal potency while other low molecular weight compounds such as rufuslactone, enokipodim, cloratin A, 2-aminoquinoline, and sesquiterpene rufuslactone exhibited minimal but substantial activity compared to grifolin. Cloratin was obtained from *Xylaria intracolarata* extract and displayed inhibition potential against *Aspergillus niger* and *C. albicans* [89, 100]*.* Phenolic acids and associated molecular compounds such as cinnamic acids and *p*hydroxybenzoic established in *G. lucidum* demonstrated potential ability against *A. versicolor*, *Trichoderma viride*, *Penicillium funiculosum*, *A. ochraceus, Aspergillus fumigatus*, *A. niger*, *P. ochrochloron,* and *P. verrucosum* [100, 105]*.*

Besides the significant benefits and potential of mushrooms of medical importance, fundamental concerns regarding the safety of certain mushrooms such as *A. bisporus* have on the other hand been reported in the recent past. The concerns have revolved around their long-term consumption uncooked. Freshly obtained *A. bisporus* induced tumors in mice upon being fed uncooked for a while. However, upon being air-dried, the mushrooms were fed to mice for 500 days and no carcinogenic tumor effect was observed [89, 106]. These results should however be considered carefully, and further laboratory research conducted to establish the certainty of these claims, since edible and medicinal mushrooms have been considered of great medical significance for centuries among many communities globally.

## Molecular pathological epidemiology of CRC research

Understanding the interactions between tumor molecular alterations and exogenous and endogenous variables can help with the identification of tumor molecular markers in CRC [107]. Microsatellite instability (MSI), CpG island methylator phenotype (CIMP), somatic *BRAF* and *KRAS* mutations, and molecular characterisation of malignancies have revealed evidence of distinct CRC subtypes that arise through activation of several neoplastic pathways. Epidermal growth factor receptor (EGFR) signalling pathway is constitutively activated as a result of *KRAS* and *BRAF* oncogene mutations [107, 108]. More lately, advanced technologies have allowed further characterisation by detecting altered genes in CRC, such as next-generation sequencing (NGS) as used in The Cancer Genome Atlas (TCGA). Epigenetics serves as a link between cellular reactions, pathogenic processes, and exogenous (environmental) factors [109]. A defining characteristic of complex multifactorial disorders is aberrant epigenetic markers (including neoplasms and malignancies such as CRC). Epigenetic signatures (DNA methylation, mRNA and microRNA expression) may function as biomarkers for risk assessment, CRC diagnosis, early detection, as well as therapeutic and chemo-preventive approaches [107, 109]. An initial phase in the development of CRC is *KRAS* oncogene mutation, which has a significant impact on colonic polyp growth and preclinical tumors [110]. Substantial evidence points to *KRAS* mutation's predictive significance in metastatic CRC treated with anti-EGFR targeted therapy [111, 112]. The IgG1 chimeric monoclonal antibody cetuximab, which targets the EGFR, is efficacious against EGFR-positive CRC tumors [112].

Edible mushrooms have been identified as potential EGRF inhibitors [113]. After some time in clinical use, EGFR tyrosine kinase inhibitors develop resistance due to mutation [113, 114]. Thelephoric acid, kynapcin-9, boletopsin-B, and physcione are naturally occurring compounds in the mushrooms *Polyozellus multiplex, Sarcodon imbricatus*, and *Cortinarius purpurascens*, respectively, and have been demonstrated to inhibit EGFR activation in molecular docking, molecular dynamics, and in Absorption, Distribution, Metabolism and Excretion (ADME) studies [113–116].

Tyrosine kinase inhibitors (TKIs) that target *EGFR* and downstream pathways frequently encounter resistance, making it more important than ever to find compounds that may be used in combination with these treatments to give cancer patients a long-lasting response [117, 118]. It has been observed that the potential of *G. lucidum* extract has significant promising efficacy in this regard [118]. To target *EGFR* and HER2, a pharmaceutical strategy utilizing tiny tyrosine kinase inhibitors (TKIs) (erlotinib and lapatinib) has been created. While lapatinib suppresses the function of *EGFR* and HER2-TK activity, erlotinib targets ATP-binding sites to prevent EGFR-TK activity [117–120]. Both of these TKIs block the downstream biological signals that encourage tumor cell survivability and proliferation from activation [118].

## Conclusion

Medicinal mushrooms are potential anti-colorectal cancer and antimicrobial agents if explored as chemo-preventive pharmaceutical products because they exhibit robust active potency. From our extensive literature search across different authentic and recognized databases, they have not been reported to exhibit adverse effects compared to synthetic chemotherapies currently in use. Tapping into the wealth of knowledge underlying the use of both edible and medicinal mushrooms in treating various infections across different communities in the world, points out their significant value in human health and wellbeing and should therefore not be ignored. Their demonstrated powerful bioactive metabolites in this research elucidate their inherent potential as suitable sources of the anticarcinoma and antimicrobial explorative pharmaceutical venture.

In addition, it is fundamental to integrate the information now available on the molecular characteristics of tumors and to define new molecular subtypes of CRC by detecting acquired somatic mutations utilizing targeted sequencing of important cancer genes. To further our understanding of the underlying carcinogenic mechanisms that underlie CRC-associations with known risk factors, it is pivotal to look at inherited genetic variation as well as lifestyle, dietary, and environmental risk factors in relation to various CRC tumor subtypes. We underscore that improving prognosis in CRC patients with *EGFR* overexpressing tumors may be best accomplished by combining treatment modalities. A focus of interest should be the development of novel and prospective *EGFR* inhibitors from edible and medicinal mushrooms and their progenitor mushrooms considering this updated review. We however recommend further research to understand the specific mechanisms of action poised by the metabolites, inhibiting, or promoting metabolic and signalling pathways that would otherwise result in CRC carcinogenesis. We further recommend research to evaluate the potential of these active phytochemicals to reach and cross the biological membrane as this will shed more light on their possible bioavailability or their unfortunate elimination from the human body system. Finally, Medicinal mushrooms should be considered as alternative natural chemo-preventive agents in the global fight against CRC and the evolution of drug-resistant pathogenic microorganisms.

## Data Availability

The datasets generated during and/or analysed during the current study are available from the corresponding author on reasonable request.
